# Supplementary material for: BOLD signal changes can oppose oxygen metabolism across the human cortex
Source: Nat Neurosci. 2025 Dec 16;29(5):1225–36. doi: 10.1038/s41593-025-02132-9 (PMC13156032; doi:10.1038/s41593-025-02132-9)
Supplement: Supplementary file 2 — Reporting Summary [file 41593_2025_2132_MOESM2_ESM.pdf]

nature portfolio

Corresponding author(s): Valentin Riedl

Last updated by author(s): October 7, 2025

Reporting Summary

Nature Portfolio wishes to improve the reproducibility of the work that we publish. This form provides structure for consistency and transparency in reporting. For further information on Nature Portfolio policies, see our Editorial Policies and the Editorial Policy Checklist.

Statistics

For all statistical analyses, confirm that the following items are present in the figure legend, table legend, main text, or Methods section.

n/a	Confirmed	
☐	☒	The exact sample size (*n*) for each experimental group/condition, given as a discrete number and unit of measurement
☐	☒	A statement on whether measurements were taken from distinct samples or whether the same sample was measured repeatedly
☐	☒	The statistical test(s) used AND whether they are one- or two-sided<br>*Only common tests should be described solely by name; describe more complex techniques in the Methods section.*
☐	☒	A description of all covariates tested
☐	☒	A description of any assumptions or corrections, such as tests of normality and adjustment for multiple comparisons
☐	☒	A full description of the statistical parameters including central tendency (e.g. means) or other basic estimates (e.g. regression coefficient) AND variation (e.g. standard deviation) or associated estimates of uncertainty (e.g. confidence intervals)
☐	☒	For null hypothesis testing, the test statistic (e.g. F, t, r) with confidence intervals, effect sizes, degrees of freedom and P value noted<br>*Give P values as exact values whenever suitable.*
☒	☐	For Bayesian analysis, information on the choice of priors and Markov chain Monte Carlo settings
☒	☐	For hierarchical and complex designs, identification of the appropriate level for tests and full reporting of outcomes
☐	☒	Estimates of effect sizes (e.g. Cohen's *d*, Pearson's *r*), indicating how they were calculated

Our web collection on statistics for biologists contains articles on many of the points above.

Software and code

Policy information about availability of computer code

Data collection	Main study: 3T Philips Elition Ingenia MR scanner (Philips Healthcare, Best, The Netherlands). Control study: 3T Philips Ingenia MR scanner (Philips Healthcare, Best, The Netherlands). Replication study: 3T Philips Elition Ingenia MR scanner (Philips Healthcare, Best, The Netherlands).
Data analysis	The calculation of the quantitative parameter maps was performed with in-house scripts in Matlab and SPM12 (Wellcome Trust Centre for Neuroimaging, UCL, London, UK), scripts can be downloaded here: https://gitlab.lrz.de/nmrm_lab/public_projects/mq-bold. The BOLD fMRI localizer data were pre-processed using fMRIPrep 20.2.4 in a docker container, which is based on Nipype 1.6.1. FMRIPrep relies on FSL 5.0.9 and ANTs 2.3.3. The subsequent data analyses and processing was performed using Python (version 3.8) and the following packages: Pandas (version 1.5.2), Numpy (version 1.23.5) and SciPy (version 1.9.3); the neuroimaging data were handled using Nilearn (version 0.9.2 and Nibabel (version 4.0.2); the plotting was performed with Matplotlib and (version 3.6.2, Seaborn (version 0.12.1). Cluster analysis was performed via atlasreader (version 0.1.2) and partial least squares analyses via pyls (version 0.1.6), permutation testing and t-test via scipy (version 1.9.3). Regression models were calculated via pingouin (version 0.5.4) and statsmodels (version 0.14.1). T<br>The scripts (Python Jupyter notebooks) for replication of all analyses and figures are available on GitHub (https://github.com/NeuroenergeticsLab/two_modes_of_hemodynamics).

For manuscripts utilizing custom algorithms or software that are central to the research but not yet described in published literature, software must be made available to editors and reviewers. We strongly encourage code deposition in a community repository (e.g. GitHub). See the Nature Portfolio guidelines for submitting code & software for further information.

Data

Policy information about availability of data

All manuscripts must include a data availability statement. This statement should provide the following information, where applicable:

- Accession codes, unique identifiers, or web links for publicly available datasets
- A description of any restrictions on data availability
- For clinical datasets or third party data, please ensure that the statement adheres to our policy

All raw and processed data are available in the online repository of OpenNeuro (Epp et al., 2023) (https://openneuro.org/datasets/ds004873).

Research involving human participants, their data, or biological material

Policy information about studies with human participants or human data. See also policy information about sex, gender (identity/presentation), and sexual orientation and race, ethnicity and racism.

Reporting on sex and gender	The sex of the participants was self-reported. 22 women and 18 men were included in the main study cohort (N=40), 11 women and 7 men were included in the control study cohort and 5 men and 5 women were included in the replication study. We did not perform separated sex-based analyses as the ratio in the main cohort was well-balanced.
Reporting on race, ethnicity, or other socially relevant groupings	We did not include reportings of race, ethnicity or other socially relevant groupings in our analyses.
Population characteristics	Three cohorts of self-reported right-handed healthy participants were analyzed. Main study cohort: 40 participants, 22 female, 18 male, mean age 32.1 ± 9.2y. Control study cohort: 18 participants, 11 female, 7 male, mean age 28.1 ± 4.8y. Replication study cohort: 10 participants, 5 female, 5 male, mean age 31.8 ± 6.8y.
Recruitment	The participants were recruited word-of-mouth and using advertisement in the newsletter from hospital and university.
Ethics oversight	Participants were informed about the objectives and potential risks of the study, and signed a written consent form. The study was approved by the local institutional review board of the Klinikum rechts der Isar (382/18S) and was conducted in accordance with the Declaration of Helsinki.

Note that full information on the approval of the study protocol must also be provided in the manuscript.

Field-specific reporting

Please select the one below that is the best fit for your research. If you are not sure, read the appropriate sections before making your selection.

☒ Life sciences ☐ Behavioural & social sciences ☐ Ecological, evolutionary & environmental sciences

For a reference copy of the document with all sections, see nature.com/documents/nr-reporting-summary-flat.pdf

Life sciences study design

All studies must disclose on these points even when the disclosure is negative.

Sample size	The sample size of healthy participants for the main study (47 subjects acquired, 40 included in the analyses) was not predetermined, as we did not find previous studies employing a cognitive design looking at ΔCMRO2. However, in a pilot study (Epp et al., OHBM poster 2019), we did find significant metabolic activation in visual cortex using visual checkerboard stimulation and mqBOLD imaging with N=12 sample size, which made us confident to identify activations in higher-order cortices with more complex designs in N=40.
Data exclusions	Seven datasets (out of 47) had to be excluded because of a) apparent behavioral task difficulties (n=1), b) problems with contrast-agent application (n=2), or c) data quality problems (severe motion artifacts in CBF or R2' data, unilateral CBF data in one condition, huge susceptibility artifacts) (n=4).
Replication	Prior to uploading our scripts on Github we cleaned all scripts and replicated all analyses and results. Further, to undermine our findings, we acquired a replication dataset with 10 subjects, employing an adjusted voxel resolution in the fMRI BOLD and BOLD ASL sequences.
Randomization	Participants were not divided into groups, so no randomization was necessary. Task conditions were displayed in a pseudo-random order, so that for every participant, every condition preceeded every other condition at least once. The fMRI BOLD task localizer was run in the beginning of the experiment for 23 subject, and at the end of the experiment for the other 17 subjects, to account for possible habituation effects that impact the BOLD-signal amplitude.
Blinding	Blinding was not relevant for this study as participants were not divided into groups.

Reporting for specific materials, systems and methods

We require information from authors about some types of materials, experimental systems and methods used in many studies. Here, indicate whether each material, system or method listed is relevant to your study. If you are not sure if a list item applies to your research, read the appropriate section before selecting a response.

Materials & experimental systems

n/a	Involved in the study
☒	☐ Antibodies
☒	☐ Eukaryotic cell lines
☒	☐ Palaeontology and archaeology
☒	☐ Animals and other organisms
☒	☐ Clinical data
☒	☐ Dual use research of concern
☒	☐ Plants

Methods

n/a	Involved in the study
☒	☐ ChIP-seq
☒	☐ Flow cytometry
☐	☒ MRI-based neuroimaging

Plants

Seed stocks	n/a
Novel plant genotypes	n/a
Authentication	n/a

Magnetic resonance imaging

Experimental design

Design type	Cognitive task design. fMRI BOLD: block design.
Design specifications	fMRI BOLD: 30s blocks, 4 repetitions per condition. Quantitative fMRI measurements: one condition per sequence, sequence durations of approx. 6min.
Behavioral performance measures	Button presses were collected during task performance in the scanner. Reaction times were analyzed for the calculation, the memory and the control conditions. Correct responses were analyzed for the calculation task and the control task.<br>To assess the validity of the memory condition, during a 15-25min break after the quantitative measurements, the 30 participants who underwent all four conditions additionally completed a memory questionnaire, where they had to indicate how easy they found it to remember concrete events (difficulty scale; 1='very easy', 2='rather easy', 3='rather difficult', 4='very difficult') and how detailed their memories were overall (concreteness scale; 1='very detailed', 2='rather detailed', 3='rather vague', 4='very vague'). On average, people scored 1.8 +/- 0.7 (mean +/- standard deviation) on the difficulty scale and 2.0 +/- 0.6 on the concreteness scale.

Acquisition

Imaging type(s)	functional fMRI BOLD, structural T1- / T2- / and T2* weighted imaging, blood flow assessment via pulsed arterial spin labeling (ASL), blood volume (CBV) assessment via dynamic susceptibility contrast imaging (contrast agent: gadolinium)
Field strength	3 Tesla
Sequence & imaging parameters	A) Multi-echo spin-echo T2 mapping: 3D gradient spin echo (GRASE), 8 echoes with even-spaced echo times (TE): TE1 = ΔTE = 16ms; TR=251; α=90°; voxel size 2x2x3.3mm3; 35 slices (30 slices in 4 subjects); total acquisition time = 2:28min (for 35 slices)<br>B) Multi-echo gradient-echo T2* mapping: 12 echoes, TE1 = ΔTE = 5ms; TR=2229ms; α=30°; voxel size 2x2x3mm3; gap 0.3mm; 35 slices (30 slices in 4 subjects). Total acquisition time = 6:08min (for 35 slices).<br>C) Dynamic susceptibility imaging (DSC): Single-shot GRE-EPI; EPI factor 49; 80 dynamics; TR = 2.0s; α=60°; acquisition voxel size 2x2x3.5mm3; 35 slices (30 slices in 4 subjects). Injection of gadolinium-based contrast agent as a bolus after 5 dynamics, 0.1ml/kg, minimum 6ml, maximum 8ml per injection (i.e. 16ml for two injections in a row, corresponding to a full clinical dosage), flow rate: 4ml/s, additionally flushing with 25ml NaCl; total acquisition time = 2:49min (for 35

slices).
E) Pseudo-continuous arterial spin labeling (pCASL):Post-labeling delay 1800ms, label duration 1800ms; 4 background suppression pulses; 2D EPI readout; TE=11ms; TR=4500ms; α=90°; 20 slices (16 slices in one subject); EPI factor 29; acquisition voxel size 3.28x3.5x6.0mm3; gap 0.6mm; 30 dynamics including a proton density weighted M0 scan; total acquisition time = 6:00min.
F) The BOLD fMRI task-localizer was acquired using single-shot EPI, EPI factor 43; voxel size = 3.0x3.0x3.0mm3; FOV 192x192x127.8mm3; TE=30ms; TR=1.2s; α=70°; 400 dynamic scans plus two dummy scans; 40 slices; SENSE-factor = 2; MB-SENSE-factor = 2; total acquisition time: 8:05min. For susceptibility correction, a B0 field map was acquired with two echoes; TR/TE1/TE2=525ms/6.0ms/9.8ms; 40 slices; parallel acquisition; α=60°; voxel size = 3.0x3.0.3.0mm3; FOV 192x192x127.8mm3; total acquisition time: 0:35s.

Area of acquisition

Whole-brain scan

Diffusion MRI ☐ Used ☒ Not used

Preprocessing

Preprocessing software

The preprocessing and calculation of the quantitative parameter maps was performed with in-house scripts in Matlab and SPM12 (Wellcome Trust Centre for Neuroimaging, UCL, London, UK).
The BOLD fMRI localizer data were pre-processed using fMRIPrep 20.2.4 (Esteban, 2019) in a docker container, which is based on Nipype 1.6.1. This included segmentation, estimation of motion-parameters and other confounds, susceptibility distortion correction, co-registration in native T1w space and normalization to MNI152 ICBM 2mm Non-linear 6th Generation Asymmetric Average Brain Stereotaxic Registration Model (Montreal Neurological Institute, McGill University). FMRIPrep relies on FSL 5.0.9 with boundary-based registration (BBR) to register BOLD fMRI EPI time series to T1w data, FSL FAST for brain tissue segmentation and spatial normalization to standard space using ANTs 2.3.3 registration in a multiscale, mutual-information based, nonlinear registration scheme, concatenating all transforms and applying all registration steps at once. This final normalization matrix to MNI 2mm space was then applied to all quantitative mqBOLD parameter maps, after co-registering all images to native T1w space. Further, as a control for partial least squares analysis described below, classical general linear model (GLM) task-analysis was implemented following recommendations in (Esteban et al., 2020).

Normalization

All data were normalized via ANTs 2.3.3 registration in a multiscale, mutual-information based, nonlinear registration scheme, concatenating all transforms and applying all registration steps at once.

Normalization template

MNI152 ICBM 2mm Non-linear 6th Generation Asymmetric Average Brain Stereotaxic Registration Model

Noise and artifact removal

The fMRI BOLD data were corrected for head motions and bias field via fMRIPrep. For task fMRI BOLD GLMs, we used CSF and white-matter signal, dvars, framewise-displacement and translations and rotations in x-, y- and z-axis as confounds, high-pass filter of 100s and 6mm smoothing.
To exclude voxels from artifact affected brain areas, we calculated the temporal signal-to-noise ratio (tSNR) from the fMRI BOLD images per subject and voxel in standard 2mm space. We then masked out voxels that were in the lowest 15th percentile in over 66% of participants and used only masked images as an input for PLS.
For the analyses in native space, we additionally used native-space parameter maps to mask CSF-influenced areas (T2 > 90ms), susceptibility-influenced areas (R2’ > 9 s-1), voxels with a high percentage of blood volume (CBV >10%, probably driven by larger veins/arteries) and voxels with T2’ > 90ms, OEF > 0.9, CBF > 90ml/100g/min.

Volume censoring

n/a

Statistical modeling & inference

Model type and settings

General linear modelling on the fMRI BOLD data, first level analysis, was applied to derive brain masks in native space. In the native space analyses, we then calculated median values within each native-space ROI (output of the first level GLM, thresholded with z>3.1) per subject and tested significant differences in task compared to baseline via paired-samples two-sided t-tests, across subjects. All other statistics were based on median voxel values within a group mask, derived from partial least squares analyses in standard space, by taking the across-subjects median value per voxel in task and in baseline. Significance in parameter values between different regions was tested via independent sample permutation test (2000 permutations) on the median voxel values.

Effect(s) tested

General linear modelling, first level analysis, was applied to derive brain masks in native space. In the native space analyses, we we then tested if the quantitative parameters values changed significantly across conditions within these ROIs.
In standard space, group masks were derived via PLS analysis. We then empirically described the distribution of these voxels separately for positive and negative fMRI BOLD ROIs.
We used regression analyses to calculate Pearson's r (regression coefficient) for different classes of voxels.
We used multiple linear regression to determine the influence of each parameter (CBF, OEF and CBF) on the change in CMRO2, as well as on baseline CMRO2. Significance of the model was determined via F-tests.

Specify type of analysis: ☐ Whole brain ☐ ROI-based ☒ Both

Anatomical location(s)

Anatomical locations were derived via comparison to Yeo et al. (2011) seven functional networks.

Statistic type for inference
(See Eklund et al. 2016)

Voxel-wise inference via partial least squares analysis on the fMRI BOLD data, see below

Correction	Group masks were derived via partial least squares analysis, see below, a multivariate analysis that does not rely on multiple comparison correction.

Models & analysis

n/a	Involved in the study
☒	☐ Functional and/or effective connectivity
☒	☐ Graph analysis
☐	☒ Multivariate modeling or predictive analysis

Multivariate modeling and predictive analysis	Mean-centered PLS is a data-reduction method that computes latent variables and corresponding brain patterns, which optimally relate brain signals to the experimental design, contrasting e.g. groups or conditions. We used PLSto get whole-brain significant clusters that best differentiate between the conditions. We used PLS for both the fMRI BOLD data as well as the quantitative parameter maps.<br>The significance of the latent variables, i.e., the entire multivariate pattern, was tested via permutation tests (3000 permutations), the reliability of the brain saliences, i.e., the voxel's contribution to the latent variables, was deduced via bootstrap resampling (3000 samples). Brain regions showing significant effects were identified via the ratio of the brain saliences to the bootstrap standard error (BSR), where a BSR > ±2 is akin to a confidence interval of 95%, if the bootstrap distribution is normal. The statistical maps were thresholded with a BSR of ±2 and only clusters with more than 30 voxels were kept for creating group masks.
